# Supplementary material for: Pathogenicity and Genomic Characterization of a Novel Genospecies, Bacillus shihchuchen, of the Bacillus cereus Group Isolated from Chinese Softshell Turtle (Pelodiscus sinensis)
Source: Int J Mol Sci. 2023 Jun 1;24(11):9636. doi: 10.3390/ijms24119636 (PMC10254083; doi:10.3390/ijms24119636)
Supplement: Supplementary file 1 [file ijms-24-09636-s001.zip › supplematary table S4. Statistics analysis of phylogenetic tree.pdf]

Table S4. Statistics analysis of phylogenetic tree

Requested genomes	36
Genomes with data	36
Max allowed deletions	0
Max allowed duplications	0
Single-copy genes found	290
Num protein alignments	290
Alignment program	mafft
Protein alignment time	483.9 seconds
Num aligned amino acids	71644
Num CDS alignments	290
Num aligned nucleotides	214932
Branch support method	RAxML Fast Bootstrapping
RAxML likelihood	-2200700.6963
RAxML version	8.2.1
