# Supplementary material for: Pathogenicity and Genomic Characterization of a Novel Genospecies, Bacillus shihchuchen, of the Bacillus cereus Group Isolated from Chinese Softshell Turtle (Pelodiscus sinensis)
Source: Int J Mol Sci. 2023 Jun 1;24(11):9636. doi: 10.3390/ijms24119636 (PMC10254083; doi:10.3390/ijms24119636)
Supplement: Supplementary file 1 [file ijms-24-09636-s001.zip › supplematary table S5. Genes used in phylogenetic tree.pdf]

Table S5. Gene used in phylogenetic tree and their statistics

PGFam	Align. Score	Align. Length	Num Seqs	Mean Sqr Freq	Prop Gaps	Product
PGF_02704551	33.32	1205	33	0.960	0.002	DNA-directed RNA polymerase beta' subunit (EC 2.7.7.6)
PGF_10049811	26.31	842	33	0.907	0.008	Protein translocase subunit SecA
PGF_06703483	23.67	643	33	0.933	0.007	DNA gyrase subunit B (EC 5.99.1.3)
PGF_00033444	22.68	661	33	0.882	0.006	Cyclic-di-AMP phosphodiesterase GdpP
PGF_06589185	22.35	594	33	0.917	0.006	Aspartyl-tRNA synthetase (EC 6.1.1.12)
PGF_00012969	21.90	615	33	0.883	0.045	Phosphomethylpyrimidine synthase ThiC (EC 4.1.99.17)
PGF_04569524	21.56	502	33	0.962	0.000	ATP synthase alpha chain (EC 3.6.3.14)
PGF_00037588	21.42	573	33	0.895	0.012	Prolyl-tRNA synthetase (EC 6.1.1.15), bacterial type
PGF_00006245	21.37	583	33	0.885	0.019	Formate--tetrahydrofolate ligase (EC 6.3.4.3)
PGF_00827693	21.26	680	33	0.815	0.123	Long chain acyl-CoA dehydrogenase [fadN-fadA-fadE operon] (EC 1.3.8.8)
PGF_00015514	21.04	465	33	0.975	0.001	Iron-sulfur cluster assembly protein SufB
PGF_00710545	20.62	502	33	0.921	0.011	Lysyl-tRNA synthetase (class II) (EC 6.1.1.6)
PGF_03000099	20.52	524	33	0.896	0.035	Ribonuclease Y
PGF_08562657	20.39	515	33	0.899	0.016	tRNA-i(6)A37 methylthiotransferase (EC 2.8.4.3)
PGF_00413298	19.88	455	33	0.932	0.012	tRNA t(6)A37-methylthiotransferase (EC 2.8.4.5)
PGF_01137124	19.70	458	33	0.921	0.021	Phosphoglucosamine mutase (EC 5.4.2.10)
PGF_01054379	19.67	437	33	0.941	0.007	Adenylosuccinate lyase (EC 4.3.2.2) @ SAICAR lyase (EC 4.3.2.2)
PGF_02516909	19.56	433	33	0.940	0.005	Enolase (EC 4.2.1.11)
PGF_07629184	19.36	500	33	0.866	0.012	Cytosol aminopeptidase PepA (EC 3.4.11.1)
PGF_04456509	19.18	590	33	0.790	0.011	2-succinyl-5-enolpyruvyl-6-hydroxy-3-cyclohexene-1-carboxylic-acid synthase (EC 2.2.1.9)
PGF_02620298	19.13	463	33	0.889	0.002	Argininosuccinate lyase (EC 4.3.2.1)
PGF_09398028	18.92	428	33	0.915	0.007	Uncharacterized protease YrrO
PGF_00015517	18.79	437	33	0.899	0.015	Iron-sulfur cluster assembly protein SufD
PGF_01462895	18.79	445	33	0.891	0.017	Uncharacterized protein YtoI
PGF_02944756	18.52	392	33	0.935	0.004	3-ketoacyl-CoA thiolase [fadN-fadA-fadE operon] (EC 2.3.1.16)
PGF_01296054	18.50	897	33	0.618	0.224	putative ATP-dependent DNA helicase YjcD
PGF_00008334	18.19	462	33	0.846	0.037	Glutamyl-tRNA reductase (EC 1.2.1.70)

PGF_01313780	17.88	375	33	0.923	0.008	Uncharacterized peptidase YqjE
PGF_00054088	17.80	441	33	0.848	0.030	Stage IV sporulation protein B
PGF_07750515	17.73	425	33	0.860	0.006	Phosphoribosylamine--glycine ligase (EC 6.3.4.13)
PGF_00828128	17.71	362	33	0.931	0.003	Putative aminopeptidase YsdC
PGF_06566462	17.70	383	33	0.905	0.007	Epoxyqueuosine reductase (EC 1.17.99.6) QueG
PGF_00423378	17.69	428	33	0.855	0.026	Dihydroorotase (EC 3.5.2.3)
PGF_07304022	17.63	375	33	0.910	0.018	tRNA-specific 2-thiouridylase MnmA (EC 2.8.1.13)
PGF_00030640	17.60	359	33	0.929	0.011	Peptide chain release factor 1
PGF_00066502	17.49	467	33	0.809	0.031	Asparaginyl-tRNA synthetase (EC 6.1.1.22)
PGF_02619921	17.29	369	33	0.900	0.004	GTP-binding protein YqeH, required for biogenesis of 30S ribosome subunit
PGF_00412178	17.29	335	33	0.945	0.023	Branched-chain alpha-keto acid dehydrogenase, E1 component, beta subunit (EC 1.2.4.4)
PGF_00054051	17.24	411	33	0.850	0.052	Stage III sporulation protein AE
PGF_00066935	17.11	464	33	0.794	0.081	Uracil permease @ Uracil:proton symporter UraA
PGF_00048782	17.09	319	33	0.957	0.006	Ribose-phosphate pyrophosphokinase (EC 2.7.6.1)
PGF_00020147	17.06	383	33	0.872	0.037	Membrane-associated protein containing RNA-binding TRAM domain and ribonuclease PIN-domain, YacL B.subtilis ortholog
PGF_00033197	17.04	323	33	0.948	0.000	Phosphate acetyltransferase (EC 2.3.1.8)
PGF_08133003	16.93	398	33	0.849	0.036	NAD-independent protein deacetylase AcuC
PGF_03004613	16.87	481	33	0.769	0.122	Histidyl-tRNA synthetase (EC 6.1.1.21)
PGF_00018952	16.82	312	33	0.952	0.000	Malate dehydrogenase (EC 1.1.1.37)
PGF_00046403	16.80	448	33	0.794	0.036	RNA polymerase sigma-54 factor RpoN
PGF_00416683	16.79	334	33	0.919	0.006	Catabolite control protein A
PGF_00907539	16.77	335	33	0.916	0.014	Branched-chain alpha-keto acid dehydrogenase, E1 component, alpha subunit (EC 1.2.4.4)
PGF_03788463	16.72	394	33	0.843	0.028	Cystathionine beta-lyase (EC 4.4.1.8)
PGF_02788759	16.63	385	33	0.848	0.013	Cysteine desulfurase (EC 2.8.1.7), associated with tRNA 4-thiouridine synthase
PGF_03677192	16.62	330	33	0.915	0.003	Lipoate-protein ligase A
PGF_02454442	16.56	382	33	0.847	0.016	N-acetyl-L,L-diaminopimelate deacetylase (EC 3.5.1.47)
PGF_00033289	16.42	334	33	0.898	0.012	Phosphate:acyl-ACP acyltransferase PlsX (EC 2.3.1.n2)
PGF_02617708	16.40	369	33	0.854	0.011	A/G-specific adenine glycosylase (EC 3.2.2.-)

PGF_03029062	16.31	314	33	0.920	0.018	Dihydroorotate dehydrogenase (NAD(+)), catalytic subunit (EC 1.3.1.14)
PGF_04139053	16.31	361	33	0.858	0.044	Uroporphyrinogen III decarboxylase (EC 4.1.1.37)
PGF_01867628	16.11	345	33	0.867	0.015	Heat-inducible transcription repressor HrcA
PGF_00696203	16.00	366	33	0.836	0.100	Peptide chain release factor 2 @ programmed frameshift-containing
PGF_00056316	16.00	376	33	0.825	0.056	Tetraprenyl-beta-curcumene synthase (EC 4.2.3.130)
PGF_04146410	15.96	364	33	0.836	0.026	Protein-arginine kinase McsB (EC 2.7.14.1)
PGF_00007027	15.95	306	33	0.912	0.016	GTP-binding protein Era
PGF_00048331	15.88	326	33	0.880	0.016	Rhodanese domain protein UPF0176, Firmicutes subgroup
PGF_00007844	15.86	331	33	0.872	0.015	Glucokinase (EC 2.7.1.2)
PGF_00033237	15.80	319	33	0.885	0.007	Phosphate starvation-inducible protein PhoH, predicted ATPase
PGF_09112178	15.69	364	33	0.823	0.050	Threonylcarbamoyl-AMP synthase (EC 2.7.7.87) / SUA5 domain with internal deletion
PGF_08005402	15.68	322	33	0.874	0.017	FIG002813: LPPG:FO 2-phospho-L-lactate transferase like, CofD-like
PGF_04797267	15.67	274	33	0.947	0.007	Naphthoate synthase (EC 4.1.3.36)
PGF_00015510	15.65	261	33	0.969	0.002	Iron-sulfur cluster assembly ATPase protein SufC
PGF_00760084	15.62	308	33	0.890	0.003	Cystathionine beta-synthase (EC 4.2.1.22)
PGF_00014295	15.53	366	33	0.812	0.021	Inner spore coat protein CotH
PGF_00015259	15.47	290	33	0.908	0.016	ATP synthase gamma chain (EC 3.6.3.14)
PGF_10097367	15.42	313	33	0.872	0.012	Porphobilinogen deaminase (EC 2.5.1.61)
PGF_10556008	15.37	410	33	0.759	0.027	ABC transporter, permease protein EscB
PGF_00008780	15.29	377	33	0.788	0.022	Glycine oxidase ThiO (EC 1.4.3.19)
PGF_00423618	15.26	300	33	0.881	0.001	Dipicolinate synthase subunit A
PGF_00048840	15.25	313	33	0.862	0.015	Ribosomal protein L11 methyltransferase
PGF_00049827	15.23	294	33	0.888	0.014	SSU rRNA (adenine(1518)-N(6)/adenine(1519)-N(6))-dimethyltransferase (EC 2.1.1.182)
PGF_00026453	15.20	292	33	0.890	0.025	Acetyl-coenzyme A carboxyl transferase beta chain (EC 6.4.1.2)
PGF_00026863	15.20	278	33	0.911	0.000	Octanoate-[acyl-carrier-protein]-protein-N-octanoyltransferase, LipM (EC 2.3.1.181)

PGF_00048928	15.15	298	33	0.878	0.016	Ribosome small subunit biogenesis RbfA-release protein RsgA
PGF_03742550	15.04	418	33	0.736	0.053	Cell division topological determinant MinJ
PGF_01751397	14.99	343	33	0.809	0.042	Adenine-specific methyltransferase (EC 2.1.1.72)
PGF_02177520	14.94	255	33	0.935	0.011	RNA polymerase sporulation specific sigma factor SigF
PGF_01456294	14.90	288	33	0.878	0.021	3-hydroxybutyryl-CoA dehydrogenase (EC 1.1.1.157)
PGF_00046551	14.85	239	33	0.961	0.001	RNA polymerase sporulation specific sigma factor SigE
PGF_06823736	14.83	273	33	0.897	0.032	2',3'-cyclic-nucleotide 2'-phosphodiesterase, Bsub YmdB
PGF_00413295	14.80	319	33	0.828	0.041	tRNA pseudouridine(55) synthase (EC 5.4.99.25)
PGF_05318435	14.79	257	33	0.923	0.006	Electron transfer flavoprotein, beta subunit
PGF_04213876	14.74	266	33	0.904	0.020	Pantothenate kinase type III, CoaX-like (EC 2.7.1.33)
PGF_00046553	14.69	291	33	0.861	0.091	RNA polymerase sporulation specific sigma factor SigG
PGF_00020984	14.57	287	33	0.860	0.027	Methenyltetrahydrofolate cyclohydrolase (EC 3.5.4.9) / Methylenetetrahydrofolate dehydrogenase (NADP+) (EC 1.5.1.5)
PGF_00008542	14.54	273	33	0.880	0.006	Glycerol uptake facilitator protein
PGF_00038971	14.54	291	33	0.852	0.044	Pur operon repressor PurR
PGF_00011463	14.40	285	33	0.853	0.040	RNA binding methyltransferase FtsJ like
PGF_00417233	14.39	354	33	0.765	0.087	L-Ala--D-Glu endopeptidase
PGF_09753067	14.34	332	33	0.787	0.042	Uncharacterized membrane protein YqjA
PGF_02366581	14.32	265	33	0.880	0.006	Transmembrane component of general energizing module of ECF transporters
PGF_01675591	14.28	271	33	0.868	0.007	Putative phosphatase YitU
PGF_03475877	14.28	288	33	0.841	0.023	Chromosome (plasmid) partitioning protein ParB
PGF_00853991	14.28	294	33	0.833	0.031	Diaminopimelate epimerase (EC 5.1.1.7)
PGF_05377575	14.27	250	33	0.903	0.012	ABC transporter, ATP-binding protein EcsA
PGF_00053849	14.24	348	33	0.763	0.044	Endospore coat-associated protein YutH
PGF_05595534	14.23	297	33	0.825	0.042	DNA polymerase I 5'-3' exonuclease domain
PGF_05930156	14.18	294	33	0.827	0.023	Inner membrane protein YihY, formerly thought to be RNase BN
PGF_00007560	14.07	384	33	0.718	0.090	Germination (Cortex hydrolysis) and sporulation protein GerM
PGF_00048586	14.06	252	33	0.886	0.027	Ribonuclease PH (EC 2.7.7.56)
PGF_00060487	14.01	255	33	0.877	0.026	Translocation-enhancing protein TepA

PGF_00205497	13.97	291	33	0.819	0.005	Uncharacterized protein YhaX
PGF_04828114	13.85	288	33	0.816	0.039	Quinolinate phosphoribosyltransferase [decarboxylating] (EC 2.4.2.19)
PGF_00040334	13.81	244	33	0.884	0.026	Alkaline phosphatase synthesis transcriptional regulatory protein PhoP
PGF_03790040	13.66	251	33	0.862	0.043	Ribonuclease III (EC 3.1.26.3)
PGF_00406573	13.55	271	33	0.823	0.023	Putative phosphotransferase YtmP
PGF_04655384	13.53	309	33	0.770	0.019	1-phosphofructokinase (EC 2.7.1.56)
PGF_00033959	13.52	227	33	0.898	0.009	Phosphoribosylformylglycinamidine synthase, glutamine amidotransferase subunit (EC 6.3.5.3)
PGF_02787474	13.45	277	33	0.808	0.030	Hydroxyethylthiazole kinase (EC 2.7.1.50)
PGF_04031671	13.33	331	33	0.733	0.089	FIG007013: polysaccharide deacetylase, putative
PGF_00689961	13.23	205	33	0.924	0.001	Guanylate kinase (EC 2.7.4.8)
PGF_10233208	13.15	256	33	0.822	0.030	16S rRNA (uracil(1498)-N(3))-methyltransferase (EC 2.1.1.193)
PGF_00014264	13.14	271	33	0.798	0.074	Inner membrane protein translocase and chaperone YidC, short form OxaI-like
PGF_06857975	13.11	228	33	0.868	0.020	Cell-division-associated, ABC-transporter-like signaling protein FtsE
PGF_02158416	13.11	231	33	0.862	0.009	Heptaprenylglyceryl phosphate synthase (EC 2.5.1.n9)
PGF_00753617	13.09	290	33	0.769	0.062	Late competence protein ComER, proline oxidase (EC 1.5.1.2)
PGF_06530721	13.04	217	33	0.885	0.002	tRNA (guanine(46)-N(7))-methyltransferase (EC 2.1.1.33)
PGF_02792560	13.01	231	33	0.856	0.005	tRNA threonylcarbamoyladenosine biosynthesis protein TsaB
PGF_00425787	13.01	229	33	0.860	0.020	FIG002379: metal-dependent hydrolase
PGF_02026136	12.97	349	33	0.694	0.180	Uncharacterized S1 RNA binding domain protein YitL
PGF_07320841	12.96	227	33	0.860	0.007	ClpCP protease substrate adapter protein MecA
PGF_00027684	12.89	215	33	0.879	0.027	Acetyltransferase AcuA, acetyl-CoA synthetase inhibitor
PGF_02629766	12.85	310	33	0.730	0.061	Sporulation sigma-E factor processing peptidase (SpoIIGA)
PGF_00039842	12.81	254	33	0.804	0.042	Putative acetyl esterase YjcH (EC 3.1.1.-)
PGF_07474537	12.76	234	33	0.834	0.051	L-cystine ABC transporter, permease protein TcyB
PGF_00048556	12.74	264	33	0.784	0.036	Ribonuclease HII (EC 3.1.26.4)

PGF_03379955	12.68	254	33	0.795	0.016	Transcriptional repressor of the fructose operon, DeoR family
PGF_01673335	12.66	276	33	0.762	0.022	2-succinyl-6-hydroxy-2,4-cyclohexadiene-1-carboxylate synthase (EC 4.2.99.20)
PGF_01072023	12.57	341	33	0.681	0.053	Allophanate hydrolase 2 subunit 2 (EC 3.5.1.54)
PGF_00413189	12.57	245	33	0.803	0.041	tRNA (adenine(22)-N(1))-methyltransferase (EC 2.1.1.217)
PGF_03462401	12.56	305	33	0.719	0.142	Peptidoglycan-N-acetylmuramic acid deacetylase PdaA
PGF_00002451	12.54	227	33	0.832	0.032	N-acetylglucosaminyl-L-malate N-acetyl hydrolase
PGF_00414889	12.46	215	33	0.850	0.005	CBS domain protein AcuB
PGF_00048926	12.44	185	33	0.915	0.000	Ribosome recycling factor
PGF_00066139	12.44	233	33	0.815	0.033	Uracil-DNA glycosylase, family 1 (EC 3.2.2.27)
PGF_00049893	12.32	235	33	0.804	0.144	SSU ribosomal protein S4p (S9e) @ SSU ribosomal protein S4p (S9e), zinc-independent
PGF_00045930	12.30	222	33	0.825	0.031	Pyrophosphatase PpaX (EC 3.6.1.1)
PGF_01753631	12.29	234	33	0.803	0.043	Uncharacterized membrane zinc metalloprotease YwhC
PGF_08122432	12.27	232	33	0.806	0.025	2-C-methyl-D-erythritol 4-phosphate cytidylyltransferase (EC 2.7.7.60)
PGF_03034879	12.22	274	33	0.738	0.074	Cell division protein FtsQ
PGF_09402727	12.18	231	33	0.801	0.042	Molybdenum ABC transporter permease protein ModB
PGF_00420087	12.18	176	33	0.918	0.000	DUF402 family nucleoside diphosphatase
PGF_04710902	12.16	260	33	0.754	0.026	Lactam utilization protein LamB
PGF_03295678	12.15	196	33	0.868	0.025	CDP-diacylglycerol--glycerol-3-phosphate 3-phosphatidyltransferase (EC 2.7.8.5)
PGF_00004058	12.11	214	33	0.828	0.088	Fatty acid metabolism regulator protein FadR, TetR family
PGF_00019428	12.09	185	33	0.889	0.038	ATP-dependent protease subunit HslV (EC 3.4.25.2)
PGF_01213071	12.03	166	33	0.934	0.000	LSU ribosomal protein L10p (P0)
PGF_06111020	12.02	214	33	0.822	0.026	Thymidylate kinase (EC 2.7.4.9)
PGF_00066124	11.99	195	33	0.859	0.075	Pyrimidine operon regulatory protein PyrR
PGF_06407625	11.96	201	33	0.843	0.015	16S rRNA (guanine(1207)-N(2))-methyltransferase (EC 2.1.1.172)
PGF_00057440	11.89	221	33	0.800	0.106	Transcription factor FapR
PGF_03751076	11.84	219	33	0.800	0.022	Riboflavin synthase eubacterial/eukaryotic (EC 2.5.1.9)
PGF_01382519	11.80	188	33	0.861	0.040	Ribosome hibernation promoting factor Hpf

PGF_06626131	11.80	160	33	0.933	0.012	2-C-methyl-D-erythritol 2,4-cyclodiphosphate synthase (EC 4.6.1.12)
PGF_00413533	11.75	169	33	0.904	0.017	Thiol peroxidase, Tpx-type (EC 1.11.1.15)
PGF_03888286	11.68	269	33	0.712	0.092	FMN reductase (NADPH) (EC 1.5.1.38)
PGF_00049453	11.65	245	33	0.744	0.131	SAM-dependent methlytransferase YrrT
PGF_00072452	11.58	175	33	0.875	0.035	Uncharacterized protein YwhD
PGF_04788810	11.49	212	33	0.789	0.083	Peptidyl-tRNA hydrolase (EC 3.1.1.29)
PGF_00053890	11.47	209	33	0.793	0.031	Spore germination protein GerD
PGF_00028472	11.42	187	33	0.835	0.036	Outer spore coat protein CotE
PGF_00416025	11.38	172	33	0.868	0.001	2H phosphoesterase superfamily protein Bsu1186 (yjcG)
PGF_00033968	11.37	195	33	0.814	0.016	Phosphoribosylglycinamide formyltransferase (EC 2.1.2.2)
PGF_08843714	11.36	196	33	0.812	0.027	Septum formation protein Maf
PGF_00026615	11.36	265	33	0.698	0.040	N-acetylglutamate kinase (EC 2.7.2.8)
PGF_00049433	11.35	162	33	0.892	0.036	S-ribosylhomocysteine lyase (EC 4.4.1.21) @ Autoinducer-2 production protein LuxS
PGF_02652319	11.34	205	33	0.792	0.068	UPF0340 protein YwlG
PGF_07133621	11.30	199	33	0.801	0.054	16S rRNA (guanine(966)-N(2))-methyltransferase (EC 2.1.1.171)
PGF_00030776	11.29	211	33	0.778	0.046	Acyl-ACP:1-acyl-sn-glycerol-3-phosphate acyltransferase (EC 2.3.1.n4)
PGF_10470343	11.26	159	33	0.893	0.052	Ferric uptake regulation protein FUR
PGF_00413203	11.21	162	33	0.881	0.024	tRNA (cytidine(34)-2'-O)-methyltransferase (EC 2.1.1.207)
PGF_00022467	11.19	174	33	0.848	0.028	Molybdenum cofactor biosynthesis protein MoaB
PGF_06649360	11.19	199	33	0.793	0.040	Segregation and condensation protein B
PGF_00408670	11.15	157	33	0.890	0.024	UPF0756 membrane protein YtwI
PGF_03889414	11.13	235	33	0.726	0.100	Uncharacterized protein YuiC
PGF_10123167	11.05	175	33	0.835	0.024	2-amino-4-hydroxy-6-hydroxymethyldihydropteridine pyrophosphokinase (EC 2.7.6.3)
PGF_07808527	11.04	202	33	0.777	0.109	ADP-ribose pyrophosphatase (EC 3.6.1.13)
PGF_00012652	11.02	187	33	0.805	0.089	FIG001553: Hydrolase, HAD subfamily IIIA
PGF_06180597	11.01	145	33	0.914	0.024	LSU ribosomal protein L13p (L13Ae)
PGF_03889881	10.94	154	33	0.882	0.009	Transcriptional regulator CtsR
PGF_00054042	10.94	171	33	0.836	0.023	Stage III sporulation protein AB

PGF_00049837	10.93	131	33	0.955	0.014	SSU ribosomal protein S11p (S14e)
PGF_00031568	10.92	148	33	0.897	0.029	Peroxide stress regulator PerR, FUR family
PGF_01043799	10.89	182	33	0.807	0.093	Transcription elongation factor GreA
PGF_00426932	10.81	171	33	0.827	0.101	6,7-dimethyl-8-ribityllumazine synthase (EC 2.5.1.78)
PGF_00011953	10.76	146	33	0.890	0.016	Histidine triad (HIT) nucleotide-binding protein, similarity with At5g48545 and yeast YDL125C (HNT1)
PGF_10546429	10.74	175	33	0.812	0.057	ATP synthase F0 sector subunit b (EC 3.6.3.14)
PGF_08432396	10.71	200	33	0.757	0.064	Nicotinate-nucleotide adenylyltransferase (EC 2.7.7.18)
PGF_05117700	10.70	168	33	0.825	0.054	Free methionine-(R)-sulfoxide reductase, contains GAF domain
PGF_00016346	10.66	120	33	0.974	0.000	LSU ribosomal protein L17p
PGF_01442793	10.64	186	33	0.780	0.031	Putative manganese efflux pump MntP
PGF_03753407	10.63	162	33	0.835	0.029	tRNA threonylcarbamoyladenosine biosynthesis protein TsaE
PGF_00020361	10.63	159	33	0.843	0.024	Metal-dependent hydrolase YbeY, involved in rRNA and/or ribosome maturation and assembly
PGF_07480521	10.59	182	33	0.785	0.133	23S rRNA (pseudouridine(1915)-N(3))-methyltransferase (EC 2.1.1.177)
PGF_08363070	10.57	161	33	0.833	0.009	Glutathione peroxidase (EC 1.11.1.9) @ Thioredoxin peroxidase (EC 1.11.1.15)
PGF_10380727	10.57	259	33	0.657	0.093	Allophanate hydrolase 2 subunit 1 (EC 3.5.1.54)
PGF_00053863	10.53	224	33	0.704	0.080	Spore cortex biosynthesis protein
PGF_00421071	10.51	147	33	0.867	0.026	D-aminoacyl-tRNA deacylase (EC 3.1.1.96)
PGF_10054809	10.45	149	33	0.856	0.022	Transamidase GatB domain protein
PGF_01447607	10.42	139	33	0.884	0.015	Uncharacterized CoA-binding protein YneT
PGF_00325052	10.38	548	33	0.443	0.394	Stage VI sporulation protein D
PGF_00267685	10.27	199	33	0.728	0.086	Sporulation initiation phosphotransferase B (Spo0B)
PGF_00007489	10.20	187	33	0.746	0.102	UPF0478 protein YtxG
PGF_10049472	10.18	181	33	0.757	0.153	Lipoprotein signal peptidase (EC 3.4.23.36)
PGF_01676425	10.16	136	33	0.871	0.040	Alkaline shock protein
PGF_00413554	10.13	186	33	0.743	0.169	tmRNA-binding protein SmpB
PGF_00049840	10.04	121	33	0.913	0.023	SSU ribosomal protein S13p (S18e)
PGF_00016445	10.00	123	33	0.902	0.030	LSU ribosomal protein L7p/L12p (P1/P2)
PGF_05636025	9.99	121	33	0.908	0.029	FIG001802: Putative alkaline-shock protein

PGF_00730798	9.95	137	33	0.850	0.055	Transcriptional regulator GlnR
PGF_01465362	9.81	182	33	0.727	0.213	Mn-dependent transcriptional regulator MntR
PGF_00064587	9.79	163	33	0.767	0.044	Uncharacterized N-acetyltransferase BT9727_3663 (EC 2.3.1.-)
PGF_09654405	9.73	175	33	0.735	0.042	FIG009886: phosphoesterase
PGF_01730118	9.66	131	33	0.844	0.030	1,4-dihydroxy-2-naphthoyl-CoA hydrolase (EC 3.1.2.28) in menaquinone biosynthesis
PGF_01935566	9.66	185	33	0.710	0.056	Nicotinamidase (EC 3.5.1.19)
PGF_03145797	9.62	125	33	0.860	0.023	UPF0382 membrane protein YwdK
PGF_01366266	9.56	164	33	0.746	0.101	Ribosomal-protein-S18p-alanine acetyltransferase (EC 2.3.1.128)
PGF_04978890	9.46	103	33	0.933	0.012	LSU ribosomal protein L21p
PGF_03681316	9.46	135	33	0.814	0.057	Hemoglobin-like protein HbO
PGF_03802161	9.40	208	33	0.652	0.167	DNA-directed RNA polymerase delta subunit (EC 2.7.7.6)
PGF_00057198	9.38	117	33	0.867	0.028	Anti-sigma F factor antagonist
PGF_03884666	9.33	171	33	0.713	0.126	Uncharacterized protein YkyB
PGF_00002047	9.27	117	33	0.857	0.009	Uncharacterized protein YojF
PGF_09452281	9.25	130	33	0.811	0.101	Ribosome-binding factor A
PGF_01979294	9.16	129	33	0.806	0.070	Dihydroneopterin aldolase (EC 4.1.2.25)
PGF_00421807	9.08	131	33	0.793	0.106	DNA replication intiation control protein YabA
PGF_03755656	9.05	164	33	0.707	0.130	Uncharacterized membrane protein YuiD
PGF_00049901	8.88	96	33	0.906	0.002	SSU ribosomal protein S6p
PGF_07836795	8.87	96	33	0.906	0.012	Aspartyl-tRNA(Asn) amidotransferase subunit C (EC 6.3.5.6) @ Glutamyl-tRNA(Gln) amidotransferase subunit C (EC 6.3.5.7)
PGF_00594236	8.82	101	33	0.877	0.020	Uncharacterized DUF1805-containing protein YunC
PGF_00419414	8.72	95	33	0.895	0.020	UPF0358 protein YlaN
PGF_00000286	8.68	108	33	0.835	0.062	FIG007421: forespore shell protein
PGF_00422085	8.66	118	33	0.797	0.176	DNA-binding protein SpoVG
PGF_08051111	8.65	88	33	0.922	0.010	Phosphotransferase system, phosphocarrier protein HPr
PGF_03011298	8.65	201	33	0.610	0.259	ComK regulator YlbF
PGF_00048932	8.47	95	33	0.869	0.048	Ribosome-associated heat shock protein implicated in the recycling of the 50S subunit (S4 paralog)
PGF_10323639	8.44	79	33	0.949	0.012	NifU-like domain protein

PGF_00142062	8.42	101	33	0.837	0.051	Uncharacterized protein YqfC
PGF_00641077	8.37	115	33	0.780	0.058	FIG139598: Potential ribosomal protein
PGF_02838109	8.33	216	33	0.567	0.213	Shikimate kinase I (EC 2.7.1.71)
PGF_00057451	8.30	111	33	0.788	0.051	Transcription regulator CDS_ID OB0894
PGF_00049858	8.29	80	33	0.927	0.035	SSU ribosomal protein S18p @ SSU ribosomal protein S18p, zinc-independent
PGF_07086362	8.26	93	33	0.857	0.019	YlxP-like protein
PGF_00056802	8.13	112	33	0.769	0.102	Thioredoxin-like protein YdbP
PGF_00015820	8.13	168	33	0.627	0.235	KapB, lipoprotein required for KinB pathway to sporulation
PGF_00007479	8.12	149	33	0.665	0.215	General stress protein 13
PGF_05313742	8.07	78	33	0.914	0.012	Uncharacterized membrane protein YuzA
PGF_00415631	7.96	96	33	0.812	0.069	COG2740: Predicted nucleic-acid-binding protein implicated in transcription termination
PGF_02454577	7.95	91	33	0.834	0.071	SSU ribosomal protein S20p
PGF_03021159	7.91	103	33	0.779	0.150	UPF0296 protein YlzA
PGF_04481164	7.87	91	33	0.825	0.061	Veg protein
PGF_00014566	7.84	141	33	0.660	0.150	Sporulation protein SpoVIF (YjcA)
PGF_10456344	7.81	77	33	0.890	0.000	Protein translocase membrane subunit SecG
PGF_00054049	7.81	129	33	0.688	0.186	Stage III sporulation protein AD
PGF_10301671	7.79	213	33	0.534	0.442	Programmed cell death toxin YdcE
PGF_10447161	7.76	74	33	0.902	0.033	Transcriptional regulator, DeoR family
PGF_08424570	7.75	160	33	0.613	0.263	Ribonuclease P protein component (EC 3.1.26.5)
PGF_06103095	7.63	89	33	0.809	0.066	Catabolite repression HPr-like protein Crh
PGF_00314829	7.51	105	33	0.733	0.120	Uncharacterized protein YtzH
PGF_00054046	7.50	68	33	0.909	0.014	Stage III sporulation protein AC
PGF_07639995	7.49	83	33	0.822	0.024	Uncharacterized protein YlbE
PGF_02011114	7.46	95	33	0.766	0.066	UPF0223 protein YktA
PGF_06916058	7.41	71	33	0.879	0.013	Uncharacterized S4 RNA-binding-domain protein YbcJ
PGF_04034430	7.35	57	33	0.973	0.007	SSU ribosomal protein S21p
PGF_00309959	7.29	73	33	0.853	0.051	Small, acid-soluble spore protein I
PGF_00721110	7.28	90	33	0.767	0.094	Uncharacterized protein YhdB
PGF_05223465	7.27	63	33	0.916	0.015	Uncharacterized protein Bsu2741.5

PGF_12808897	7.24	63	33	0.913	0.031	2-hydroxymuconate tautomerase-like protein YwhB (Bsu3751)
PGF_05076104	7.23	86	33	0.780	0.119	Exodeoxyribonuclease VII small subunit (EC 3.1.11.6)
PGF_06429692	7.19	69	33	0.866	0.057	Uncharacterized protein CAC3725
PGF_04163874	7.02	90	33	0.740	0.130	hypothetical protein
PGF_00685189	7.01	108	33	0.674	0.207	Protein translocase subunit YajC
PGF_05831938	6.87	61	33	0.879	0.041	Small acid-soluble spore protein SspF
PGF_05906360	6.85	70	33	0.818	0.006	DNA-directed RNA polymerase omega subunit (EC 2.7.7.6)
PGF_00225029	6.81	77	33	0.776	0.131	Uncharacterized transcriptional regulator YazB, Cro/CI family
PGF_00147658	6.73	63	33	0.848	0.049	Uncharacterized protein YfjT
PGF_02054288	6.58	71	33	0.781	0.068	Uncharacterized protein YqgQ
PGF_02645954	6.54	96	33	0.667	0.158	UPF0298 protein YlbG
PGF_00054075	6.48	134	33	0.560	0.326	Stage III sporulation protein D
PGF_00053296	6.01	50	33	0.850	0.021	Small, acid-soluble spore protein O
PGF_09316422	5.98	122	33	0.541	0.278	UPF0213 protein YazA
PGF_00345520	5.87	58	33	0.771	0.131	Sporulation inhibitor sda
PGF_07467475	5.46	52	33	0.757	0.112	hypothetical protein
