# Supplementary material for: Pathogenicity and Genomic Characterization of a Novel Genospecies, Bacillus shihchuchen, of the Bacillus cereus Group Isolated from Chinese Softshell Turtle (Pelodiscus sinensis)
Source: Int J Mol Sci. 2023 Jun 1;24(11):9636. doi: 10.3390/ijms24119636 (PMC10254083; doi:10.3390/ijms24119636)
Supplement: Supplementary file 1 [file ijms-24-09636-s001.zip › supplematary table S8 QF108-045 antigenicity prediction.pdf]

Supplementary Table S8. Application of genome sequencing facilitated to identify genes associated with virulence, disease and defense in *B. cereus* genome. The table represent classification of virulent products their subcellular localization, antigenicity score and solubility.

Classification	Gene	Annotation	Location of Gene	Product	PSORTb	Antigenicity	Predicted Solubility upon Overexpression
Enzyme	inhA (orf02552)	VFDB	Chromosome	Immune inhibitor A metalloproteinase	Extracellular	0.95	SOLUBLE with probability 0.97
Enzyme	piplc	VFDB	Chromosome	Phosphatidylinositol-specific phospholipase C (PI-PLC)	Extracellular	0.89	SOLUBLE with probability 0.76
Enzyme	SMase (orf02211)	VFDB	Chromosome	Sphingomyelinase	Extracellular	0.91	SOLUBLE with probability 0.62
Immune evasion	bpsA (orf00255)	VFDB	plasmid	*B. cereus* exo-polysaccharide (BPS)	CytoplasmicMembrane	0.71	SOLUBLE with probability 0.64
	bpsC (orf003720)	VFDB	Chromosome	*B. cereus* exo-polysaccharide (BPS)	Cytoplasmic	0.55	SOLUBLE with probability 0.61
	bpsD (orf00248)	VFDB	plasmid	*B. cereus* exo-polysaccharide (BPS)	CytoplasmicMembrane	0.18	SOLUBLE with probability 0.84
	HasA	VFDB	plasmid	Hyaluronic acid (HA) capsule	CytoplasmicMembrane	0.25	INSOLUBLE with probability 0.61

	PC (orf03337)	RAST	Chromosome	Polysaccharide capsule	CytoplasmicMembrane	0.88	INSOLUBLE with probability 0.63
	Mobile element Org124	RAST	plasmid	Mobile element protein	Extracellular	0.85	INSOLUBLE with probability 0.86
	Type II SS Orf39	RAST	plasmid	type II secretion system protein, putative	Extracellular	0.75	INSOLUBLE with probability 0.73
	RBP Orf81	RAST	plasmid	reticulocyte binding protein	Extracellular	0.90	INSOLUBLE with probability 0.70
Toxin	cya orf00126	VFDB	plasmid	Anthrax toxin (edema factor)	Extracellular	0.60	INSOLUBLE with probability 0.82
	pagA orf00155	VFDB	plasmid	Anthrax toxin (protective antigen)	Extracellular	0.90	INSOLUBLE with probability 0.79
	alo orf05784	VFDB	Chromosome	Anthrolysin O	Extracellular	0.93	INSOLUBLE with probability 0.80
	hlyII orf05511	VFDB	Chromosome	Hemolysin II	Extracellular	0.93	INSOLUBLE with probability 0.65

	orf03096	VFDB	Chromosome	Hemolysin III homolog	CytoplasmicMembrane	0.18	INSOLUBLE with probability 0.56
	hlyIII orf00403	VFDB	Chromosome	Hemolysin III	CytoplasmicMembrane	0.19	INSOLUBLE with probability 0.79
	hblA orf05994	VFDB	Chromosome	Hemolytic enterotoxin HBL	Extracellular	0.90	INSOLUBLE with probability 0.80
	hblC orf05991	VFDB	Chromosome	Hemolytic enterotoxin HBL	CytoplasmicMembrane /Cellwall/Extracellular	0.94	INSOLUBLE with probability 0.57
	hblD orf05991	VFDB	Chromosome	Hemolytic enterotoxin HBL	Extracellular	0.93	INSOLUBLE with probability 0.55
	nheA orf00783	VFDB	Chromosome	Non-hemolytic enterotoxin (Nhe)	CytoplasmicMembrane	0.93	INSOLUBLE with probability 0.51
	Nhe orf00782	VFDB	Chromosome	Non-hemolytic enterotoxin (Nhe)	Extracellular	0.93	INSOLUBLE with probability 0.63
	Nhe orf00781	VFDB	Chromosome	Non-hemolytic enterotoxin (Nhe)	Extracellular	0.93	INSOLUBLE with probability 0.61

O-antigen	OA orf05690	VFDB	Chromosome	O-antigen	Cytoplasmic	0.55	INSOLUBLE with probability 0.71
	OA orf5691	VFDB	Chromosome	O-antigen	unknown	0.79	INSOLUBLE with probability 0.64
