# Supplementary material for: Pathogenicity and Genomic Characterization of a Novel Genospecies, Bacillus shihchuchen, of the Bacillus cereus Group Isolated from Chinese Softshell Turtle (Pelodiscus sinensis)
Source: Int J Mol Sci. 2023 Jun 1;24(11):9636. doi: 10.3390/ijms24119636 (PMC10254083; doi:10.3390/ijms24119636)
Supplement: Supplementary file 1 [file ijms-24-09636-s001.zip › supplematary table S9 virulence factor's open reading frame sequence.pdf]

contig_1=QF108-045 chromosome
contig_2=QF108-045 plasmid

>orf00658 contig_2|bacillus sp. SYJ|Bacillus|orf00658 contig_2
MKYSLCTISFRHQLISFTDIVQFAYENGFEGIELWGTHAQNLYMQEYETTERELNCLKDK
TLEITMISDYLDISLSADFEKTIEKCEQLAILANWFKTNKIRTFAGQKGSEDFSEQERKE
YVNRIRMICDLFAQHNMYVLLETHPNTLTDTLPSTLELLGEVNHPNLKINLDFLHIWESG
AKPIDSFHQLKPWIQHYHFKNISSADYLHVFEPSNVYAAAGSRTGMVPLFEGIVNYDEII
QEVRDTEHFASLEWFGNNTKDILKEEMKVLTNRNLEVVTS
>orf00659 contig_2|bacillus sp. SYJ|Bacillus|orf00659 contig_2
MYGMKVNEWYNHAKDKDSNVETFLQLVENKPENRYVIVMVDMSLLPERENKFHQKPFPHY
LMISKTEKEEEWFMLDPDFRWEGNMEREKVLHSVQDNPFGGGYFIDVEEIREPTAEMVAR
YFIETFKRDDNELTMELKNLIIKMANEEEGYSLSGLVAAVKQIPVLAIRKYSYEHAFAYF
RETLQYSEQEFDYWCDRVEDIVQGFTNVQYRAIKMAMTKNKGMLLSIVEKLDEMNAIELQ
IKEELEKQFLSWKEMKSNESVLVF
>orf00660 contig_2|bacillus sp. SYJ|Bacillus|orf00660 contig_2
MRREALKDAVLKIMTEKMELKNVTHLEETMRLNQDLYIDSVMMLQLIVYIEMDVMLCVPE
DEVDPKAFLTVGSLLDFMEELQPLQDINVNN
>orf00781 contig_2|bacillus sp. SYJ|Bacillus|orf00781 contig_2
VKNMQKRFYKKCLLAVMIAGVATSNAFPLYPFAAEQNVKVLQENVKNYSLGPAGFQDVMA
QTTSSIFAMDSYAKLIQNQQETDLSKISSINSEFKGNMIQHQRDAKINATYWLNNMKPQI
MKTDQNIINYNNTFQSYYNDMLIAIDQKDSGKLKADLEKLYADIVKNQNEVDGLLGNLKA
FRDRMAKDTNSFKEDTNQLTSILASTNAGIPALEQQINTYNDSIKKSNDMVIAGGVLCVA
LITCLAGGPMIAVAKKISQMQKEKSPI
>orf00782 contig_2|bacillus sp. SYJ|Bacillus|orf00782 contig_2
MTKKPYKVMALSALMAVFAAGNIMPAHTYAAESTVKQVPVHATAKTYNDYEEYSLGPEGL
KDAMERTGSNALVMDLYALTIIKQGNVNFGNVSTVDAALKGKVIQHQDTARGNAKQWLDV
LKPQLISTNQNIINYNTKFQNYYDTLVAAVDAKDKATLTKGLTRLSSSINENKAQVDQLV
EDLKKFRNKMTSDTQNFKGDANQITSILASQDAGIPLLQNQITTYNEAISKYNAIIIGSS
VATALGPIAIIGGAVVIATGAGTPLGVALIAGGAAAVGGGTAGIVLAKKELDNAQAEIQK
ITGQITTAQLEVAGLTNIKTQTEYLTNTIDTAITALQNISNQWYTMGSKYNSLLQNVDSI
SPNDLVFIKEDLNIAKDSWKNIKDYAEKIYAEDIKVVDTKKHN
>orf00783 contig_2|bacillus sp. SYJ|Bacillus|orf00783 contig_2
VKKTLITGLLVTAVSTSCFIPVSAYAKEGQTEVKTVYAQNVIAPNTLSNSIRMLGSQSPL
IQAYGLVILQQPDIKVNAMSSLTNHQKFAKANVREWIDEYNPKLIDLNQEMMRYSIRFNS
YYSKLYELAGNINEDEQSKADFTNAYGKLQLQVQSIQESMEQDLLELNRFKTVLDKDSNN
LSIKADEAIKTLQGSSGDIVKLREDIKRIQGEIQAELTTILNRPQEIIKGSINIGKQVFT
ITNQTAQTKTIDFVSIGTLSNEIVNAADSQTREAALRIQQKQKELLPLIQKLSQTEAEAT
QITFVEDQVSSFTELIDRQITTLETLLTDWKVLNNNMIQIQKNVEEGTYTDSSLLQKHFN
QIKKVSDEMNKQTNQFEDYVTNVEVH
>orf01408 contig_2|bacillus sp. SYJ|Bacillus|orf01408 contig_2
MKIIYDRGYYFEKNYMKALVVATTLAIPFAAYSTPALAALKAEANQSVVAASERTYATEI
KIYKDQKDEPSMVSQYIKDPKVTIAAGKKIVTVTMQDSDYFQYLRIEDRNQPGVFHDVKV
LSEDKRKNGTKVVQFEIGEFEKKHNMQMHILIPAIGYDHKYQVQFEIKDPTVGNKETEKP
DDNSNSGNTETDHPVDNQNMITDNKLRELVNKKVFNRKDLNTPITKEELLQVKDLFLNTN
EILDYSALKYMPNLKSLTVANAKIKDPSFFTSLKQLKHLALRGNEFSDVTPLVKMDHLES
LDLSNNKITNVAPLIEMKNVKSLYLSGNQIEDVTALAKMEQLDYLNLANNKITNVAPLSA
LKNVTYLTLAGNQIEDIKPLYSLPLTDLVLTRNKVKDLSGIEQMKQLEELWIGKNEIKDV
TPLSKMTQLKELHLPNNELKDITPLSSLVNLQKLDLEANYISDLTPASNLKKLVFLSFVA
NEIRDVRPVIELSKTAYINVQNQKVFLEETEVNKEVKVPIYEKDGKISTKIRLKSEGGTY
SNDAVKWSTLGEKIYEFGVKDPFADTGIFFTGSVIQNVVESQGDNTSKEDEKVEFKDVPK
GHWSEEAINYLAKENIFKGYGNGQFGFGDSITRGQVASLVQRYLKLENKVEQKERFTDTK
GHMFEQDIATVAQAGIMQGDGAGEFRPDGVLTRYEMSVVLYKVFQLKEDENNKVNFNDVP
TGHWAEGYVKALVDNNISKGDGKGKFLGDDFVTREQYAQFLYNAITK
>orf02211 contig_2|bacillus sp. SYJ|Bacillus|orf02211 contig_2
MEVWNMTGKLLKGVLSFGIGLGVLYGGSSAQADTSTDQNNTLKVMTHNVYMLSTNLYPNW
GQSERADLIGAADYIKNQDVVILNEVFDNSASDRLLGNLKREYPNQTAVLGRSNGNEWDK
TLGSYSSSTPEDGGVAIVSKWPIVEKIQYVFAKGCGPDNLSNKGFVYTKIKKNDHFVHVI
GTHLQAEDSMCGKTSPASVRTNQLKEIQDFIKNKNIPNDEYVLFGGDMNVNKINAENNSD

SEYAWMFKTLHASIPSYTGHTATWDATTNSIAKYNFPDSPAEYLDYIIASKDHANPSFIE
NKVLQPKSPKWTVTSWLKNIHIMITLIIIQ
>orf02217	contig_2|bacillus sp. SYJ|Bacillus|orf02217	contig_2
MRRKAPFKVLSSLAIAAIIGCTSVMSAPLAYAETPAKEKENVSTTPIDYNLIQEDRLAEA
LKERGAINPASSKEETKKAVEKYIEKKQGDQANKEILPADTAKEASDFVKKVKEKKMEEK
EKVKVKKPEKNVSPEQKPEPNKKQLNGQVPTSKAKQAPYKGPVRNDKVLVLLVEFSDYKH
NNIDQTPGYMYSNDFSREHYQKMLFGNEPYTLFDGSKVKTFKQYYEEQSGGSYTTDGYVT
EWLTVPGKASDYGADGSSGHDNKGPKGARDLVKEALHAAAEKGLDLSQFDQFDRYDTNGD
GNQNEPDGVIDHLMVIHAGVGQEAGGGKLGDDAIWSHRSKLAIDPVAIEGTKSKVDYFGG
KVAAHDYTIEPEDGAVGVFAHEFGHDLGLPDEYDTKYTGTGSPVEAWSLMSGGSWTGKIA
GTEPTSFSPQNKDFLQKNMGGNWAKILEVDYDKIKRGVGVPTYIDQSVTKSNRPGVVRVN
LPGKSVETIKPEFGKHAYYSTRGDDMHTTLETPFFDLTKGINAKFDYKANFELEAECDFV
EVHAVTEDGTKTLIDRLGEKVVQGDKDTTDGKWIDKSYDLSQFKGKKVKLQFDYITDPAV
TYKGFAMDNVNVTVDGQVVFSDDAEGTSNMQLNGFVVSDGTEKKAHYYYLEWRNYAGSDN
GLKAGKGPVYNTGLVVWYADDSFKDNWVGMHPGEGFLGVVDSHPEALVGNLNGKPTYGNT
GMQIADAAFSFDQTPAWSVNSFTRGQFNYSGLQGVTTFDDSKVYSNKQIADAGRKVPNLG
LKFQVVGQAEDKSAGAVWIKR
>orf03720	contig_2|bacillus sp. SYJ|Bacillus|orf03720	contig_2
VMKRVRKAIIPAAGLGTRFLPATKAMPKEMLPIVDKPTIQYIVEEAVKSGIEDIIIVTGK
TKRSIEDHFDNAFELEQNLLEKKKYELLEKVQASSKMVDIHYIRQKEPKGLGHAVWCARK
FIGDEPFAVLLGDDIVQAEKPCLRQLIEEYDKTLSSVIGVQTVPEDETHRYGIIDPLEQE
GRRYQVRNFVEKPAAGTAPSNLAIMGRYVLTPEIFMFLEQQHVGAGGEIQLTDAIQSLNE
IQRVFAYDFEGKRYDVGEKLGFVQTTIEMALQHPELRDDMVVMMKKILEEHTNQES
>orf05784	contig_2|bacillus sp. SYJ|Bacillus|orf05784	contig_2
VIFLNNKKNTKRRKFLTCVLVSLCTLNYSSISFAETQASHVTDITKNASSIDTGIGNLTY
NNQEVLAVNGDKVESFVPKESINSNGKFVVVEREKKSLTTSPVDISIIDSVANRTYPGAV
QLANKAFADNQPSLLVAKRKPLNISIDLPGMRKENTITVQNPTYGNVAGAVDDLVSTWNE
KYSTTHTLPARMQYTESMVYSKSQIASALNVNAKYLDNSLNIDFNAVANGEKKVMVAAYK
QIFYTVSAELPNNPSDLFDNSVTFGELTRKGVSNAAPPVMVSNVAYGRTVYVKLETTSKS
KDVQAAFKALLKNNSVETSGQYKDIFEESTFTAVVLGGDAKEHNKVVTKDFNEIRNIIKD
NAELSLKNPAYPISYTSTFLKDNATAAVHNNTDYIETTTTEYSSAKMTLDHYGAYVAQFD
VSWDEFTFDQNGKEVLTHKTWEGSGKDKTAHYSTVIPLPPNSKNIKIVARECTGLAWEWW
RTIINEQNVPLTNEIKVSIGGTTLYPTATISH
>orf05993	contig_2|bacillus sp. SYJ|Bacillus|orf05993	contig_2
MLKNIHLNQELSRINANYWLDTAKPQIQKTARNIVNYDEQFQNYYDTLVETVQKKDKAGL
KEGINDLITTINTNSKEVTDVIKMLQDFKGKLYQNSTDFKNNVGGPDGKGGLTAILAGQQ
ATIPQLQAEIEQLRSTQKKHFDDVLAWSIGGGLGAAILVIAAIGGAVVIVVTGGTATPAV
VGGLSALGAAGIGLGTAAGVTASKHMDSYNEISNKIGELSMKADRANQAVLSLTNAKETL
AYLYQTVDQAILSLTNIQKQWNTMGANYTDLLDNIDSMQDHKFSLIPDDLKAAKQSWNDI
HKDAEFISKDIAFKQE
>orf05995	contig_2|bacillus sp. SYJ|Bacillus|orf05995	contig_2
MMKKIPNKLLAVSAFLTITTTYAVIPLETFAIEIEQMNNENISLSANEEQMKKALQDAGL
FAKAMNEYSYLLIHNPDVSFEGITINGNADLPSKIVQDQKNARAHAVTWNTQVKKQLFDT
LTGIIEYDTKFENHYETLVEAINTGNGDTLKKGIKDLQGGIQQNQKSAKALIEELIQLKN
AIGEDVRTFGSHKETLQSILKNQGADVEADQKRLEDLLGQVKYQKDIESKGLDMVKIPFI
PTLIAGGIMIGDARGKLGWLEPELAKLRQTVDYKITLNRVVGVAFHNISDMHSMLDNAIT
ALTYMSTQWEDLDSQYSGVLGHIDKADQKADQNKYKFLTPSLNAAKNSWKTLKTDVVTLQ
EGIKIAEKKEQDFLNQLRPANVFYFYKKIHNAYTFEIKTGTNAPNASYKVMNLTKNTVHN
MWSGGANTNMWADWLSFNPNDEFAVVAVVDGKEYVVYKDKVQNIMN
>orf06015	contig_2|bacillus sp. SYJ|Bacillus|orf06015	contig_2
MKKVKDAKADAQEKVDQPAGGPVVGEAPVKGGLNGKVPTTSAKQKQYNGDVRKDKVLVLL
VEFADFKHNNIDQVPGYMYSNDFNPEHYQKMLFGDEPFTLFDGSKVPTFKQYYEEQSGGS
YTVDGTVTKWLTVPGNAAEYGADAGDGGHDNKGPKGPRDFVKEALNAAVESGIDLSEFDQ
YDQYDNNGDGNKNEPDGLIDHLMVIHAGVGQDGGGGRLGDDAIWSHRWNLGTPYPIEGTK
AKVDNWGGKMAAYDYTIEPEDGAVGVFAHEFGHDLGLPDEYDTKYSGAGEPINSWSVMSG
GSWAGKIAGTTPPSFSPQNKEYFQKNMGGNWANIVEVDYDKLNRGIGFATYLDQSVTKSN
RPGLIRVNLPDKDVKNIQPAFGKKYYYSTKGDNLHTTLETPVFDLKNATNVKFDYKTLYE
IETDYDFLEVYAVTEDDKKTLVDRIGEKNVKNGLDTTDGNWVDKSYDLSQFKGQKIKLAF
EYITDVGIAPKGFTLDNATLNVDGNVVFSDDAEGEAKLKLNGFVVSNGFEKKKHNYYLEW
RNYAGADQALKYSSGVPYNTGLVVWYADSSFTDNWVGLHPGEGFLGVVDSHPEAIVGTLN
GKPTVKNSTRYQIADAAFSFNQAPAWKVVSPTRGTYDYKGLPGVTKFDDSKSYMNDLIPD

AGRKLPKLGLKFEVVGQADDNTAGAVRLYR
>orf06980 contig_2|bacillus sp. SYJ|Bacillus|orf06980 contig_2
MAIPSISVYKMPIESELPKNKVNWTPDPKRAVLLIHDMQEYFLDAYSDKESPKVELISNI
KMIREKCKELGIPVVYTAQPGGQTLEQRGLLQDFWGDGIPAGPDKKKIVDELTPDEDDIF
ITKWRYSAFKKTNLLEILNEQGRNQLIICGIYAHIGCLLTACEAFMDGIEPFFVGDAVAD
FSLEHHKQALEYASNRCAVTTSTNLLLNDLQSVKDDESEGITIQEVHELVAQLLREPVES
IDIDEDLLNRGLDSVRIMSLVEKWRREGKEITFADLAERPTVAGWYSLLSSQTAQVL
>orf06981 contig_2|bacillus sp. SYJ|Bacillus|orf06981 contig_2
MLVGYTEWPKEFADRYREEGCWLGETFGGVLRERAEKYGDQIAVVSGKKHITYSELNKKV
DRLAAGLLNLGIKKEDRVVIQLPNIIEFFEICFALFRIGALPVFALPSHRSSEISYFCDF
GEASAYVISDKALGFDYRKLAREVKEKVPNLQHVIVVGEEEEFVNINDLYMDPVSLLEVQ
PSDVAFLQLSGGTTGLSKLIPRTHDDYIYSLRVSAKICNLNAESVYMAVLPVAHNYPMSS
PGTFGTFYAGGKVVLATGGSPDEAFALIEKEKVTITALVPPLAMIWLDAASSRNADLSSL
EVIQVGGAKFSAEVARRIRPTFGCTLQQVFGMAEGLVNYTRLDDPEEIIIHTQGRPMSAL
DEVRVVDENDNDVQPGKVGSLLTRGPYTIRGYYKAEEHNARSFTKDGFYRTGDLVKVNEQ
GYIIVEGRDKDQINRGGEKVAAEEVENHLLAHDAIHDVAIVSMPDDYLGERTCAFVIVRG
QVPAVSELKMFLRERGIAAYKIPDRIEFIESFPQTGVGKVSKKNYVKSLLKNLLQ
>orf06984 contig_2|bacillus sp. SYJ|Bacillus|orf06984 contig_2
MNLGEFDEKTVLVTGAAQGIGSVVAKMFLERGATVVAVDQNEEGLQVLLKQNELNKTRMK
TFCLDVSDSAAVEDMINHIANEIAPIDILVNVAGVLRMGPIHSLSDEDWNKTFSVNTTGV
FNMSRAVSKNMMLRKSGAIVTVGSNAANTPRMEMAAYAASKAATTMFMKCLGLELAAYNI
RCNLVSPGSTETEMQRLLWADENGAKNIIAGSQNTYRLGIPLQKIAQPSEVAEAVLFLAS
DRASHITMHNLCVDGGATLGV
>orf00403 contig_2|bacillus sp. SYJ|Bacillus|orf00403 contig_2
MTEKMTRMTQFVKEEIANAITHGIGAILSIPALIILIIHASKHGTASAVVAFTVYGVSMF
LLYLFSTLLHSIHHPKVEKLFTILDHSAIYLLIAGTYTPFLLITLRGPLGWTLLAIIWTL
AIGGIIFKIFFVRRFIKASTLCYIIMGWLIIVAIKPLYENLTGHGFSLLLAGGILYSVGA
IFFLWEKLPFNHAIWHLFVLGGSAMMFFCVLFYVLPTA
>orf01469 contig_2|bacillus sp. SYJ|Bacillus|orf01469 contig_2
MTAVLGLSFGAGTQSAYAETPVNKTATSPVDDHLIPEERLADALKKRGVIDSKASETETK
KAVEKYVENKKGENPGKEVSNGDPLTKEASDFLKKVKDAKADTKEKVDKPATGTPAATGP
VKGGLNGKVPTSPAKQKAYNGDVRKDKVLVLLVEYADFKHNNIDKEPGYMYSEDFNKEHY
EKMLFGNEPFTLDDGSKIETFKQYYEEQSGGSYTVDGTVTKWLTVPGKAADYGADAASGG
HDNKGPKGPRDLVKDALKAAVDSGIDLSEFDQFDQYDVNGDGNQNQPDGLIDHLMIIHAG
VGQEAGGGKLGDDAIWSHRWTVGPKPFPIEGTQAKVPYWGGKMAAFDYTIEPEDGAVGVF
AHEYGHDLGLPDEYDTQYSGQGEPIESWSIMSGGSWAGKIAGTTPTSFSPQNKEFFQKTI
GGNWANIVEVDYEKLNKGIGLATYLDQSVTKSNRPGMIRVNLPDKDVKTIAPAFGKQYYY
STKGDDLHTKMETPLFDLTNATTAKFDFKSLYEIEAGYDFLEVHAVTEDGKQTLIERLGE
KANSGNADSTNGKWIDKSYDLSQFKGKKVKLTFDYITDGGLALNGFALDNASLTVDGKVV
FSDDAEGTPQLKLDGFVVSNGTEKKKHNYYVEWRNYAGADNALKFARGPVFNTGMVVWYA
DSAYTDNWVGVHPGHGFLGVVDSHPEAIVGTLNGKPTVKSSTRFQIADAAFSFDKTPAWK
VVSPTRGTFTYDGLAGVPKFDDSKTYINQQIPDAGRILPNLGLKFEVVGQADDNSAGAVR
LYR
>orf02212 contig_2|bacillus sp. SYJ|Bacillus|orf02212 contig_2
MKKKVLALAAAITLVAPLQSVAFAHENDGGSKIKIVHRWSAEDKHKEGVNSHLWIVNRAI
DIMSRNTTLVKQDRVAQLNEWRTELENGIYAADYENPYYDNSTFASHFYDPDNGKTYIPF
AKQAKETGAKYFKLAGESYKNKDMKQAFFYLGLSLHYLGDVNQPMHAANFTNLSYPQGFH
SKYENFVDTIKDNYKVTDGNGYWNWKGTNPEEWIHGAAVVAKQDYSGIVNDNTKDWFVKA
AVSQEYADKWRAEVTPMTGKRLMDAQRVTAGYIQLWFDTYGDR
>orf02301 contig_2|bacillus sp. SYJ|Bacillus|orf02301 contig_2
MSQNQFECHISEEDVQMLRALAHPLRLRLVMELMQRGTCNVTQLQEVLEIPQSTVSQHLT
KLKQNKVVRFERRGLEVYYQIHNDKVSEVVKTLFS
>orf02552 contig_2|bacillus sp. SYJ|Bacillus|orf02552 contig_2
MYSDDFNQEHYQKMLFGDEPFELYDGTSIKSFKKYYEEQSGGSYTVDGTVTNWLTLPGKA
ADYGRDTDSGGRDNKGPKGPRDLVKEALQAAADSGIDLSNFDQFDQYDINGDGDRNQPDG
VIDHLMVIHAGMGQEAGGGKLGNDAIWSHRSIVSPLPYEIEGTKSKVPYWNGKIAAFDYT
IEPEDGAVGVFAHEFGHDLGLPDEYDTALENKGQGEPIAGWSLMSSGSWAGKIVGTAPTS
FSPQNKEFFQKIMGGNWVNITEVDYDKLNRGIGYATYLDQSVSKTDRPGIIRVNLPDKEV
KGIESAFGKKYYYSTKGDNLHTKLETPMFDLTKAKIVNFDFKTLYEIEMEHDLLEVHAVT
EDGTNILIDTIGKKNVTNGVDTTLGKWVDKSYDLSQFKGTKVKLVFEYMTDDSLALNGFA
LDNATLTVDGRPIFSDDAEGKPKLKLDGFTISNGIEKKKHNYYLEWRNYAGSDEALRFSQ

GPVYNTGMVVWYADSSYTDNWVGVHPGHGFLGVVDSHPKAIVGTLNGQPTFRNSTRFQVA
DAAFSFNQTLSWKVVSSKSGTFIYDGLPGVAKFDDSNTYINEQIPDAGRILPKLGLKFEV
VGQADDNTAGAIRLYR
>orf03096 contig_2|bacillus sp. SYJ|Bacillus|orf03096 contig_2
MNTYIREPVNAFTHLGGAVLSFIALLAMLVKVSIKMPSFAAITAIILFGIGMMVLYTASA
VYHSVVASERVIYFFRKLDHSMIFILIAGTYAPFCLITLHSENGLLLFCLVYATAICGIV
FKMFWFNCPRWLSTAIYITMGWLIVLFFAPLAANLSTGGMVLLVLGGILYTIGGFIYGTK
PKWLEFNHMGHHEIFHVFVLLGSLAHFLSVYCYVI
>orf03226 contig_2|bacillus sp. SYJ|Bacillus|orf03226 contig_2
MHAEKLGSEIKKIRVMRGLTQKQLSENICHQSEVSRIESGAVYPSMDILQGIAAKLQVPI
IHFYEVLVYSDIERKKLFKDRIQMLSKKKQYAEIYNIVSNELKKEKFHPEFNQFLLWYYY
LSAYVLQKINFEYCILELRKIVHHSYGGIDVFQNLYIENSIASICAENNQLDRAITSFRA
ILEQLESLHNDEAFTVKVRYNYAKSLYLSDQFEEALYQVNEAIEASRHMGSMELIGQLYY
QKGECLEKLEYSSDDIKEVYKKRLSSSIY
>orf03227 contig_2|bacillus sp. SYJ|Bacillus|orf03227 contig_2
MKKLLIGSLLTLAMAWGISLTDTALEKSQVISHNDQEVQLASDLPFEH
>orf03228 contig_2|bacillus sp. SYJ|Bacillus|orf03228 contig_2
MLTTDTWVLIGYFIAQSLVIYAVISLYRREQYSSSRISLIIGFTIILVYGYIHMFQLNAG
IKPSSEVSYLIHTASILLIGLSSILYILDKPTQHETKTKYYRFDYVRFILPYFSIIITFS
FIIFQPWDDKFMLIGLILSLILLFLRQLYMWKDNQALIDTYEQLTTQLEGKVEEGASALS
KSEQRYTSLFEDHPDAVFSLNMYGIFQQSNTACESLFTAYYCEVESYSLLHFIDSKDHDL
LKKALQLTKEGRPQTLEVRTKEKEGYYYYLHITLIPTFINKEVVGMFGIARDITTLYEKQ
KQVEHLAFHDALTGLPNRRKFEKDLKNILNTAQTSANDVAVMFLDLDRFKKINDRLGHDV
GDLLLIEVAKRLRGCLRSKDVVARQGGDEFTILLPDMYSEKSAAFIAEQILNILNKPFFI
QGEELSITPSIGIAMYPDYGTDVTELMKNADMAMYRAKANGKNRFVFFSKEMSIAQNEIQ
FLEGELAKALQQNEFFLEYQPQVSTKTKQIIGFEALIRWKHPKLGIVSPAQFIPLAEETG
FIIELGNWILRTACLEAKRWHNQGFSQLKVGVNLSVVQFNHADLIPTISKVLEETELKPE
ALDIEITESIAINQNQSVVAKLEQLQNLGIQISIDDFGTGYSSLAYLTKYPINTLKIARE
FICGITTSPLEEAIISSIITLSKELNLEVIAEGVETEEQWKFLYEQNCDHIQGFFISKPV
SSKDVWRLLHKKTTV
>orf03330 contig_2|bacillus sp. SYJ|Bacillus|orf03330 contig_2
MDDYVKPLVEEAGFTVGEDIYLVHCPERVLPGQILHELVYNNRIVGGVTPTCAEAGARVY
GVFVQGEIIKTNAKTAEMSKLMENTFRDVNIALANELTKVCNSLDINVLDVIEMANKHPR
VNLHYPGPGVGGHCLAVDPYFIVAKAPELANIIKLSRETNVSMPQYVTTQVKGLLNGIES
PKVAAFGVTYKGNVDDMRESPAMDVIDLIRNEGITVAAHDPHVESDFFELHSAEEAVKDA
HLILILTDHSEFKTLDYGKLAPSMKQALVFDTRNCVQANTDSNIKIINFGNVYEEVKGKV
VTP
>orf03337 contig_2|bacillus sp. SYJ|Bacillus|orf03337 contig_2
MKKKILFWVLGILGVLIIGGGIYAYNVYSSVSNTLKEVHQPLKRDQNNSKVGEKVSKSEP
VSILLLGADERGDDKGRSDSLMVITLNPKNNSMKTVSIPRDTYTEIVGKGKSDKINHAYA
FGGVDMSVATVENFLNVPINYYIEVNMEGFKDIVDAVDGVDVNNDLEFTQDGHHFAKGNI
HLTGDQALAFTRMRKQDPRGDFGRQMRQRQVMQGVIKKGASFSSLTGYGDVLAAIQKNVK
TNLTQDQMFDMQKNYKDCLKNSEDIQIPGDGHKAADGIWYYYVPDAAKQDLTNKLRAHLE
VTK
>orf03338 contig_2|bacillus sp. SYJ|Bacillus|orf03338 contig_2
MNSILICGGAGYIGSHAVKKLVDEGLSVVVVDNLQTGHEDAITEGAKFYNGDLRDKAFLR
DVFTQENIEAVMHFAADSLVGVSMEKPLQYYNNNVYGALCLLEVMDEFKVDKFIFSSTAA
TYGEVDVDLITEETMTNPTNTYGETKLAIEKMLHWYSQASNLRYKIFRYFNVAGATPNGI
IGEDRRPETHLIPLVLQVALGQREKIMMFGDDYNTPDGTCIRDYIHVEDLVAAHFLGLKD
LQNGGESDFYNLGNGNGFSVKEIVDAVREVTNHEIPAEVAPRRAGDPARLVASSQKAKEK
LGWDPQYVNVKTIIEHAWNWHQKKPNGYEK
>orf05511 contig_2|bacillus sp. SYJ|Bacillus|orf05511 contig_2
MKKANGIAKSVAVASVIMSGSLGLQATSAFADSKGTVENLQNGGKVYNSFKTTYDMKQNI
KNSIKVSFIEDPYADKKIAIVTTDGSNIDAKYTINGGYYNAGLKWPSAYHTEAEITSGDS
AQFHKAAPVNTMTSAKVTSEVGYTLGGSVKVGVNDKGPNADASITGSFAWKESVSYDQVD
YKTVLETHTDKKLNWKVGFQSFNYPEWGIYNRDSFNTFYGNQLFMKSRSYNEGTNNFVSK
DTYNEFFTNNYKLDWKNHQVTLDNQKALVEQMSSINNVNNQLNKGKGKLSFSMNGNQLKA
TSSNAGYGISYEDKNWGIFVNGEKVYTFNEKTTVGNISNDINKLNIKGPYIEIKQI
>orf06126 contig_2|bacillus sp. SYJ|Bacillus|orf06126 contig_2
MKKKKKLKPLAVLTTAAVLSSTFAFGSHAAYADAPPSLPIDEHLIPEERLAEALKQRGVI
DQSASQAETSKAVENYVEKKKGENPGKEILTGDNLTQEASDFMKKVKDAKMKENEQAQQP

EVGPVAGQGAGLNPGKLNGKVPTTPAKQAEYNGAVRKDKVLVLLVEFSDFKHNNIDQEPG
YMYSKDFNREHYQKMLFGDEPFTLFDGSKINTFKQYYEEQSGGSYTVDGTVTEWLTVPGK
ASDYGADAGTGHDNKGPLGPKDFVKEALKAAVAKGINLADYDQFDQYDQDGDGNKNEPDG
IIDHLMVVHAGVGQEAGGGKLKDDAIWSHRSKLGSKPYAIDGTKSSVSNWGGKMAAYDYT
IEPEDGAVGVFAHEYGHDLGLPDEYDTKYSGQGEPVESWSLMSGGSWAGKIAGTEPTSFS
PQNKEFFQKNMKGNWANIVEVDYDKLRTGIGTATYLDQSVTKSKRPGIIRVNLPDKDIKN
IESAFGKKFYYSTKGNDIHTTLETPVFDLTTATDAKFDYKAFYELEAKYDFLDVYAIAED
GTKTRIDRMGEKDVKGGADTTDGKWVDKSYDLSQFKGKK
>orf06977 contig_2|bacillus sp. SYJ|Bacillus|orf06977 contig_2
MQATPTLWQALVTDYPEKLQGLSILVGGEALPAHLANKLRELGCSITNLYGPTETTIWST
FMNIDEGEKGIPPIGKPICNTEVYVLDAGLQPVPPGAIGELYIAGEGLASGYLGKPELTA
ERFVANPYGESGKRMYRTGDLVKWRSDGALEYISRADHQIKIRGFRIELAEIETVLQRHK
NIQQAVVMVREDRANDKRIIAYIVAEEKEPINLSEIRSYVSESLANYMIPSAFVVLEELP
LTPNGKVDRKKLPAPDFNGMNNERVARNPKEEILCDLFAEVLGVSRISIDDNFFEMGGHS
LLASRLMARIRETLSVELGIGKLFESPTVAELAKQLNHAKSARPAIQKASRPNEVPLSFA
QRRLWFLNCLEGPSPTYNIPLVIRMNGILNREALQGAFYDVVEKHETLRTIFPNVLGSSY
QKILDMENLNLEMVITNTCKDELESVLSEAVRYSFNLDFEPAVRLQLFTVSENEHVLLIL
LHHIVGDGWSLQPLTRDFTAAYKARCQGDRIQLESLPVQYADYALWQQQLLGDETTPESL
ISTQLDFWKEELKGLPDQMELPTDFQRPIETSYRGETIHFHIDEGMHSRLVELARENGVS
LFMVLQAGLSALFTRLGAGTDIPIGSPIAGRNDDVLSDIVGLFVNTLVLRTNTSGDPSFK
ELLNRVKQVNLAAYENQDVPFERLVEVLNPVRTRNSHPLFQVMLAFQNTPEATFHVPDLE
ASLEIQSVGSAKFDLTFEISESNGVDGTPNGLHGLLEFSTDLYKRETVQKLIERFILLLD
DAVTNPDQSIGRLEILTLAEKNTVLEKWNGGFQIAPEMTLPQLFEKQVHINPNSIAVVFE
DKKLTYEELNRKANKIARFLIAKGIGPDQLVALAMPRSLNMVVSLLAVLKAGAGYLPLDP
DYPSDRISFMLHDAKPSCVLTNSEVEIECDEALKVLVDDVKVIAEVEKYSEDNIDEVERI
NPLSPSNIAYVIYTSGSTGRPKGVMIPHQNVVRLLGATDHWYQFDGNDVWTMFHSYAFDF
SVWEIWGPLLYGGRLVIVPHTVSRSPKEFLQLLVKEKVTVLNQTPSAFYQLMQADCENEE
IGQKLSLRYVVFGGETLELSRLEDWYSRHSHNAPKLINMYGITETTVHVSYIELDETIVS
LRANSLIGCSIPDLKVYVLDNYLQPVPPGVVGEMYVAGAGLARGYLGRAGLTAGRFIADP
FGKPGTRMYRTGDLARWRHDGTLDYIGRADHQIKIRGFRIELGEIEAVIMKHPKVEQVAV
IVREDQPGDKRLVSYIVSSNNEAIDTNEMRQFASGSLPDYMVPYAFVVANELPLTPNGKL
DRKALPAPEFIASSSSRGPRTPQEEMLCDLFTEVLSVPQIGIDDGFFDLGGHSLLAVQLM
SRMKEALGVELNIGTLFAAPTVAGLAERLEMGNGQSALDVLLPLRASGDQLPLFCVHPAG
GLSWCYAGLMKSLGTDYPIYGVQARGIAKNEELPKSLEEMAADYLKHVREVQPHGPYRLL
GWSLGGNVVHAMAAQIQNEGEEVELLVMLDSYPGHFLPNTEAPTEEEALIALLALGGYDP
DNMDGKPLTMESAVEILRKDGSALASLEEETILNLKETYVNSVGLLGKYIPKVYNGDILF
FRSTVIPDWFDPISPNTWLNYLDGQIVQHDIDCRHKDLCQPGPLTEIGQVLAKYLQNKKG
VSTV
>orf06988 contig_2|bacillus sp. SYJ|Bacillus|orf06988 contig_2
MTTYTSEMKKTLVSEEDVDILKIMAHPIRLQIVNELSTRKTCNVTQLTELLNIPQSTVSQ
HLSKMKGKVLRAERRGLEIYYHINNLKASKIVNVLGYIN
>orf01032 contig_2|bacillus sp. SYJ|Bacillus|orf01032 contig_2
MKMQTDLLNIFFEESEEHLQSLNENVLILEQNPADMDVVGEIFRSAHTFKGMSASMEFTE
MADLTHKMENVLDEIRHGNIVVHANIIDVIFECIDNLEKMVADVQQGGMGNIDVVSTKQK
LEALLNGNVETSMEHVEQESINNDDAVSHEVHITVEQQAILKAVRAIMCIEALQNIGGIQ
KTVPSIEEIEADAFGFEFTVFMNTDRSEEELKQVVLHVSEIEKVVVKQGESLQEVDSTVA
TQEDARVEEMIQPSVVAQVEAPIETVKQPVSALPTKSTAKTKNAKGENRSIRVQLEKIER
LMNMFEESVIERGRIDELAQAIQNKELIEHLNRLGDISKDIQNVLLNMRMVPIETVFNRF
PRMVRMLAKDLGKKIDLQITGEDTEVDKIVIDEIGDPLVHLIRNAIDHGIETVEQRRDAG
KNETGTIKLEAFHSGNHVVIQITDDGNGIHKGKVLEKAIKNGVVTESEANKLTDREVFDL
IFQPGFSTAEVVSDLSGRGVGLDVVKHTIHSLGGHLIIDSEEGKGSTFRIELPLTLSIIQ
SMLVQTNDKRYALPLGNIVEAIRIKREDIQSIQGKDVLNYRDQIIEVKHLSTVFGEKTVD
EAFESYESQMVPVLIVRNTHRSYGLIVNTIIGQREIVLKSLGDFFAESSNYFSGATILGD
GRVVLILNPEGL
>orf05690 contig_2|bacillus sp. SYJ|Bacillus|orf05690 contig_2
MASSSFWNKKKVFITGHTGFKGSWLTLFLTSLGAEVVGFSSHPPSIPNLFEQGNVAKECI
TIHGDITDYNSLFHALKQHNPDILFHLAAQPIVTTSYKNPIETFKTNVLGTVHVLEAAKH
IDSIRGIINVTSDKCYENDGSGNQAFVESDRLGGFDPYSASKACAELVATSYQKSFFRTN
TQKLASVRAGNVIGGGDWAEDRLFPDVIRAYLQDDTLTIRNKNAIRPWQHVLDPLHGYIL
LAEKLWTDAEYAEAWNFGPLNEPNRTVHDVIQSIIKLWNKPLTILPPLQILLMNRLF
>orf05691 contig_2|bacillus sp. SYJ|Bacillus|orf05691 contig_2

MKAVILAGGYGTRIGEETHLKPKPMIEIGTKPILWHIMSLYSHYGITEFIICLGYKGYAI
KEFFLNYNLHMSDFTIQLNDNTITSHSHRVEPWKVTLIDTGVNTETGGRVKKIQNFVGDE
PFCLTYGDGLSNVNIKELIAFHKKHGKMATVTAVQPPGRFGSLILDKQSVTSFQEKPLGD
GGWVNGGFFVLNPEVFNYISGDKSVFETDTLVQLVNKNELAAYQHTGFWHPMDTLRDKNK
LVELWESNNAPWKVW
>orf06554 contig_2|bacillus sp. SYJ|Bacillus|orf06554 contig_2
MQHILIIEDEESLADFLELELKYEGYKVDIQFDGRKGLEVALETNYDLILLDLMLPGLNG
LEVCRRLRATKNTPIIMLTARDSIMDRVTGLDSGADDYLPKPFAIEELLARMRVIFRREE
HIENKHVSSLTFKDLQLQIESRTITKGNEEMELTNKEFELLLMFMKNINRVLTRDVLLNQ
VWGYDAMVETNIVDVYVRYLRNKLHSVDKEEYIQTVRGAGYIMK
>orf02381 contig_2|bacillus sp. SYJ|Bacillus|orf02381 contig_2
MKQNKRKRINAMIIAATLSLPFAVYSTPALAAVAIEANKTGQGLEDGTYEAVIKAYKDKT
NEESMAAVYIKNPKLTIENGKKIVTATLSDSDFFQYLKTEDIHTPGVFHDVKVISEDKKK
NGTKVIQFEVGELGKRYNMQMHIYIPTMFYDNKYQVQFEVNALNLENNVPKEQKENKEEK
VEQQDESGNVILDKQLQKYINKYNLDRDNVDAPITKKDLLQIKTLSIYSGKGIREITGLE
YMTNLEKLTLRESNVKDISAISKLRGLKYVDLTSNSIESIHPIEQLENINMLF
>orf05991 contig_2|bacillus sp. SYJ|Bacillus|orf05991 contig_2
VYGMKTKIITGLLVTSIVTGGNIPINTLATPIVQAETQQEGTDISSSLRKLGAQSKLIQT
YIDQSLMSPNVQLEEVTALNTNQFLIKQDMKEWSSELYPQLILLNSKSKGFVTKFNSYYP
TLKSFVDNKEDREGFSDRLEVLQEMAMTNQENTQRQINELTDLKLQLDKKLKDFDTDVVT
AQGILSTDGTGKIDQLKNEILNTKKQFKMIYSKLH
>orf05994 contig_2|bacillus sp. SYJ|Bacillus|orf05994 contig_2
MKETLQKAGLFAKSMNAYSYMLIKNPDVNFEGITINGYVDLPGRIVQDQKNARAHAVTWD
TKVKKQLLDTLNGIVEYDTTFDNYYETMVEAINTGDGETLKEGITDLRGEIQQNQKYAQQ
LIEELTKLRDSIGHDVRAFGSNKELLQSILKNQGADVDADQKRLEEVLGSVNYYKQLESD
GFNVMKGAILGLPIIGGIIVGVARDNLGKLEPLLAELRQTVDYKVTLNRVVGVAYSNINE
MHKALDDAINALTYMSTQWHDLDSQYSGVLGHIENAAQKADQNKFKFLKPNLNAAKDSWK
TLRTDAVTLKEGIKELKVETVTPQK
>orf00110 contig_1|bacillus sp. SYJ|Bacillus|orf00110 contig_1
MKNFFINIKPYTSYEKFKSYLPKGENSLKQNKIKSALKMPFILLVFSLLIVAMYYINFTI
RGSFSLFLGIYGTLMVIYLLGKQSLSFFYRPITGDKVPNMKVAVVVPSYNESASAIVNTI
NSVLAQDYPIHEIFFVDDGSKDKSAYEVALKMREELLRTQREIAATTENNCSEILGIPDL
IVHRLPKNCGKRHAQLWAFKRTTADAIVTIDSDGDLFPNAVRELLKPFNDEKVMATTGHV
NIRNRNDNLLTKLIDMRYDNAFRVERAAQSVTGNVLVCSGPLSCYRREVITENLEHYGSQ
MFLGEEVQFGDDRCLTNYAILKGKTVYQSTARCITDAPTTLKQFLKQQLRWNKSFFRESL
ISLAIGMKKPNVLVWTIFEISLWILFGLSLLLSIILKASHVGLILAVYYLGYISLAAYAR
NVFYLLKHPLTFLLAPLYGILHVLALLPIRFYALLTIKSNGWGTR
>orf00248 contig_1|bacillus sp. SYJ|Bacillus|orf00248 contig_1
MSNTSSISRTESKDRLGYLHEKKSYLFMKRFIDIVAASFGIVLLCWLFFLLAILIKLEDP
KGPVFFKQKRIGKKEKEFYMFKFRSMVSNAEELLDSILNLNEVSGAMFKIQKDPRVTKVG
KFIRRTSIDELPQLWNVLKGDMSLVGPRPPLPREVVKYTDYDKQRLLVTPGCTGLWQVSG
RNDLSFKEMVELDIIYIKNRSVMNDLKIIIKTFKIIFSPNGAY
>orf00249 contig_1|bacillus sp. SYJ|Bacillus|orf00249 contig_1
VLEKVQASSKINIHYIRQKEPKGLGHAVWCARKFIGNEPFAVLLGDDIVQAETPCLRQLM
DEYEGTQSAVIGVQKVPENEVHRYGIIDPVEQNNRRYQVRQFVEKPAEGTAPSNLAIMGR
YVLTPEIFMFLENQQTGAGGEIQLTDAIQRLNEIQSVFAYDFEGTRFDVGEKLGFIKTTI
EMALQDKGIQEELLNHMEWLLKSESSREYK
>orf00255 contig_1|bacillus sp. SYJ|Bacillus|orf00255 contig_1
MIFKRLNSDQEVYNPIELQTNLQLINTYNEMITSYNILEKVVKNLNLNETVEELISKVNI
KNEKNSQVITILVQDKNPKQAAAVANEISRVFRDEIISIMNLDNVTVLTKADATGEQIPV
KPQPLLNIIVALVIGLFVGVVISFLMEYSDKTIRSEKDVMDLLELSVLGSIATNSNTINS
RKFKTKKRPFRGGTID
>orf00162 contig_1|bacillus sp. SYJ|Bacillus|orf00162 contig_1
MTVFVDHKIEYMSLEEDADLLKVMAHPMRLKLVNELYKHKVLNVTQLIQILKLPQSTVSQ
HLSKMRGKVLKGNRQGLEIYYSINNPKIERIIELLSPIQ
>orf00225 contig_1|bacillus sp. SYJ|Bacillus|orf00225 contig_1
MTTIQASNEMYKIPEADVELLKIMAHPVRLQIVKELEHRKICNVTQLTELLDVPQSTVSQ
HLSKMRGKILRSERRGLEMYYHIANSKACQIVSVLGL
>orf00126 contig_1|bacillus sp. SYJ|Bacillus|orf00126 contig_1
MKKEGMEKDRIDVLKGEKALKASGLVPEHADTFKKIARELNTYILFRPVNKLATNLIKSG
VATKGLNVHGKSSDWGPVAGYIPFDQDLSKKHGQQLAVEKGNLENKKSITEHEGEIGKIP

LKLDHLRIEELKENGIILKGKEEIDNGKKYYLLESNNQVYEFRISDENNEVQYKTKEGKI
TVLGEKLNWRNIEVMAKNVEGVLKPLTADYDLFALAPSLTEIKNKYHKKNGIK
>orf00155 contig_1|bacillus sp. SYJ|Bacillus|orf00155   contig_1
MALSTILVSNTSNLGVIQAEVTQENQLLNESESSSQGLLGYYFSDLNFQEPMLITPSTTG
DLSISSSELENIPSENQYFQSAIWSGFIKVEKSEEYTFATSTDDHVTMWVDNQEVINKSS
NSNKIRLEKGQLYQIKIQYQRENPTEKGLDFKLYWTDSQNKKKLFLVITYNCQN
