# Supplementary material for: Pathogenicity and Genomic Characterization of a Novel Genospecies, Bacillus shihchuchen, of the Bacillus cereus Group Isolated from Chinese Softshell Turtle (Pelodiscus sinensis)
Source: Int J Mol Sci. 2023 Jun 1;24(11):9636. doi: 10.3390/ijms24119636 (PMC10254083; doi:10.3390/ijms24119636)
Supplement: Supplementary file 1 [file ijms-24-09636-s001.zip › supplementary figures.pdf]

(A)

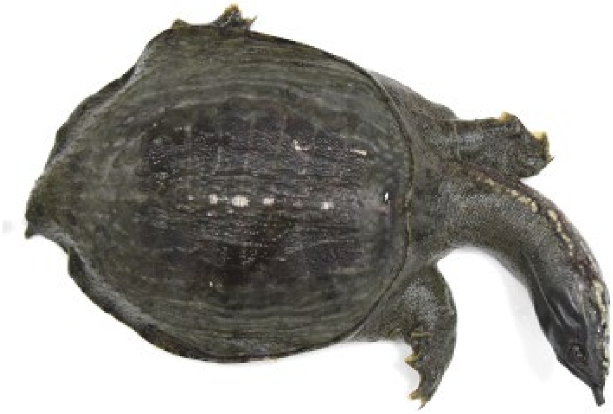

(B)

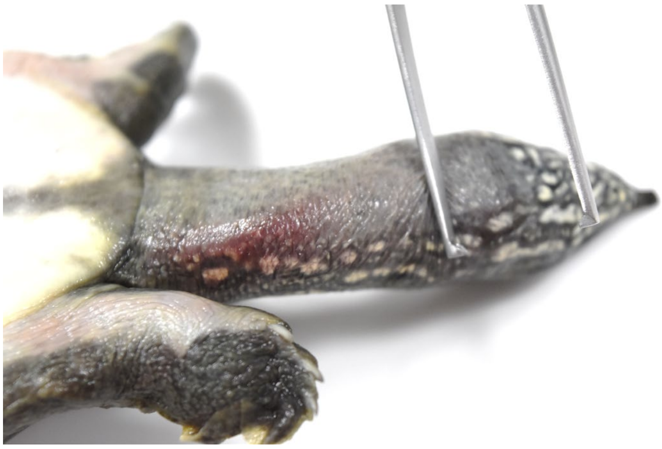

(C)

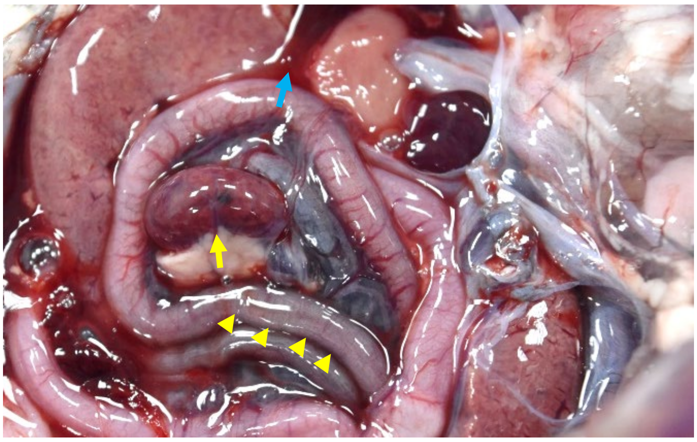

(D)

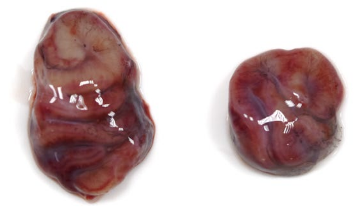

Figure S1 Gross lesion of *Bacillus shihchuchen* biovar anthracis QF108-045 challenged CST. (A) Neck opisthotonus, (B) skin haemorrhages, (C) serosanguineous fluid accumulation (blue arrow), intestinal serosa oedema (yellow arrowhead), and splenomegaly (yellow arrow), (D) kidney haemorrhage.

(A)

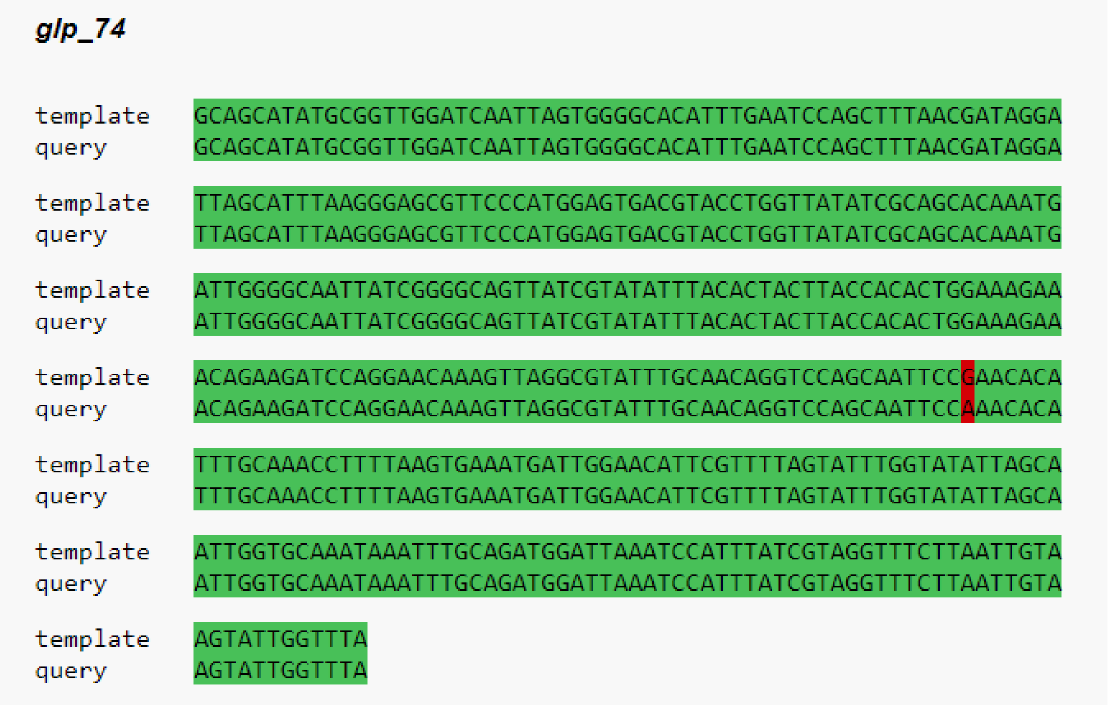

(B)

pta_21

```
template    AATTTAACATTAGCAGGCGTTGATATTTACGACCCAGCTACATACGAAGAAATGGATGCA
query       AATTTAACATTAGCAGGCGTTGATATTTACGACCCAGCTACATACGAAGAAATGGATGCA

template    ATGGTAGCATCTTTCGTTGAACGCCGTAAAGGTAAAGCAACTGAAGAAGACGCTCGCAAA
query       ATGGTAGCATCTTTCGTTGAACGCCGTAAAGGTAAAGCAACTGAAGAAGACGCTCGCAAA

template    ATCCTTAAAGACGAAAACTACTTCGGTACAATGCTTGTATACATGGGCAAAGCACACGGT
query       ATCCTTAAAGACGAAAACTACTTCGGTACAATGCTTGTATACATGGGTAAAGCACACGGT

template    TTAGTAAGTGGTGCAGCTCACTCTACAGCTGATACAGTTCGTCCAGCACTTCAAATTATT
query       TTAGTAAGTGGTGCAGCTCACTCTACAGCTGATACAGTTCGTCCAGCACTTCAAATTATT

template    AAAACAAAACCAGGCGTTACAAAAACTTCTGGCGTATTCATCATGGTACGTGAAGAAGAG
query       AAAACAAAACCAGGCGTTACAAAAACTTCTGGCGTATTCATCATGGTACGTGAAGAAGAG

template    AAATATGTATTCGCTGATTGCGCAATTAACATTGCACCAAACAGCCAAGATTTAGCTGAA
query       AAATATGTATTCGCTGATTGCGCAATTAACATTGCACCAAACAGCCAAGATTTAGCTGAA

template    ATTGGTATCGAGAGTGCGAAAACTGCTGAACTATTCGGCATTGACCCACGCGTT
query       ATTGGTATCGAGAGTGCGAAAACTGCTGAACTATTCGGCATTGACCCACGCGTT
```

Figure S2. Two loci (Glp and Pta) in *Bacillus shihchuchen* biovar anthracis QF108-045 genome had two novel alleles, which their sequence were shown in (A) Glp, and (B) Pta.
